# Preparing for patients with high-consequence infectious diseases: Example of a high-level isolation unit

**DOI:** 10.1371/journal.pone.0264644

**Published:** 2022-03-03

**Authors:** Frieder Pfäfflin, Miriam Songa Stegemann, Katrin Moira Heim, Stephan Achterberg, Ursula Pfitzner, Louise Götze, Lars Oesterhelweg, Norbert Suttorp, Christian Herzog, Benjamin Stadtmann, Alexander Uhrig

**Affiliations:** 1 Department for Infectious Diseases and Respiratory Medicine, Charité–Universitätsmedizin Berlin, Corporate Member of Freie Universität Berlin, Humboldt-Universität zu Berlin, Berlin Institute of Health, Berlin, Germany; 2 Institute of Legal Medicine and Forensic Sciences, Charité–Universitätsmedizin Berlin, Corporate Member of Freie Universität Berlin, Humboldt-Universität zu Berlin, Berlin Institute of Health, Berlin, Germany; 3 Centre for Biological Threats, Strategy and Incident Response, Robert Koch-Institute, Berlin, Germany; University of Hail, SAUDI ARABIA

## Abstract

**Introduction:**

Patients with high-consequence infectious diseases (HCID) are rare in Western Europe. However, high-level isolation units (HLIU) must always be prepared for patient admission. Case fatality rates of HCID can be reduced by providing optimal intensive care management. We here describe a single centre’s preparation, its embedding in the national context and the challenges we faced during the severe acute respiratory syndrome coronavirus type 2 (SARS-CoV-2) pandemic.

**Methods:**

Ten team leaders organize monthly whole day trainings for a team of doctors and nurses from the HLIU focusing on intensive care medicine. Impact and relevance of training are assessed by a questionnaire and a perception survey, respectively. Furthermore, yearly exercises with several partner institutions are performed to cover different real-life scenarios. Exercises are evaluated by internal and external observers. Both training sessions and exercises are accompanied by intense feedback.

**Results:**

From May 2017 monthly training sessions were held with a two-month and a seven-month break due to the first and second wave of the SARS-CoV-2 pandemic, respectively. Agreement with the statements of the questionnaire was higher after training compared to before training indicating a positive effect of training sessions on competence. Participants rated joint trainings for nurses and doctors at regular intervals as important. Numerous issues with potential for improvement were identified during post processing of exercises. Action plans for their improvement were drafted and as of now mostly implemented. The network of the permanent working group of competence and treatment centres for HCID (Ständiger Arbeitskreis der Kompetenz- und Behandlungszentren für Krankheiten durch hochpathogene Erreger (STAKOB)) at the Robert Koch-Institute (RKI) was strengthened throughout the SARS-CoV-2 pandemic.

**Discussion:**

Adequate preparation for the admission of patients with HCID is challenging. We show that joint regular trainings of doctors and nurses are appreciated and that training sessions may improve perceived skills. We also show that real-life scenario exercises may reveal additional deficits, which cannot be easily disclosed in training sessions. Although the SARS-CoV-2 pandemic interfered with our activities the enhanced cooperation among German HLIU during the pandemic ensured constant readiness for the admission of HCID patients to our or to collaborating HLIU. This is a single centre’s experience, which may not be generalized to other centres. However, we believe that our work may address aspects that should be considered when preparing a unit for the admission of patients with HCID. These may then be adapted to the local situations.

## Introduction

A high-consequence infectious disease (HCID) is defined as an acute infectious disease, which typically has a high case-fatality rate, may not have effective prophylaxis or treatment, may have the ability to spread in the community and within healthcare settings, and requires an enhanced population and system response to ensure it is managed effectively, efficiently and safely [1]. Public Health England lists, as of now, 16 diseases as HCID [1]. HCID occur rarely in Western Europe and the United Kingdom. There is experience of managing cases of Ebola virus disease (EVD), Lassa fever, Marburg virus disease (MVD), Crimean Congo hemorrhagic fever (CCHF), Middle East respiratory syndrome (MERS), severe acute respiratory syndrome (SARS), and monkeypox [1–4]. The majority of these patients acquired their infections overseas, but rare incidents of secondary transmission have occurred for EVD and Lassa fever [5, 6]. The diagnosis of HCID may be challenging for several reasons. First, HCID are rare and physicians are unfamiliar with their clinical presentations. Second, a detailed travel history is often omitted in busy clinical routine. Third, early signs and symptoms of HCID are usually non-specific. Once the diagnosis of a HCID has been established, the respective patient is transferred to a high-level isolation unit (HLIU).

In 2009, a framework for the design and operation of HLIU was published by the European Network of Infectious Diseases (EUNID) [7]. It defines important prerequisites for patient care, occupational health, diagnostic services, transport, air handling, and environmental hygiene. However, a cross-sectional analysis in sixteen countries found that adherence to recommendations varied substantially, both within and between different countries [8]. EUNID also developed a three-day curriculum for training healthcare workers in the management of HCID comprising theoretical knowledge and practical skills [9]. Although such a curriculum can help to standardize training contents, it can neither address the need for continuously refreshing skills nor the features that may be unique to a single centre. A team from the Johns Hopkins University has described the creation of a biocontainment unit [10] and the unit’s impact beyond HCID preparedness in detail [11]. Numerous issues are addressed but these experiences do not explicitly focus on staff preparation.

In Germany, the permanent working group of competence and treatment centres for HCID (Ständiger Arbeitskreis der Kompetenz- und Behandlungszentren für Krankheiten durch hochpathogene Erreger (STAKOB) at the Robert Koch-Institute (RKI)) is in charge of the management of patients with HCID. It encompasses experts for public health preparedness and response from eight German centres and experts for clinical management of patients with HCID from seven German tertiary care hospitals with HLIU. One additional hospital and several of the aforementioned hospitals also serve as training centres. Coordination of the working group is facilitated by an office at the RKI, which is the German government’s central scientific institution in the field of biomedicine and one of the most important bodies for safeguarding public health in Germany. STAKOB members regularly meet twice yearly. In addition, experts meet virtually online whenever required to discuss case management and procedures. However, STAKOB does not define the preparation at the individual HLIU in detail, which is necessary to effectively handle patients with HCID. This issue has been discussed on several occasions but conditions vary greatly between centres and thus, a uniform approach was not feasible.

To the best of our knowledge, the preparation process for the admission of HCID patients has not yet been described with a focus on the medical team. Moreover, the current situation was complicated by the SARS-CoV-2 pandemic. We here describe how the preparation for patients with HCID is handled in our HLIU and the impact of the SARS-CoV-2 pandemic on the preparation process.

## Methods

### Department and HLIU

**Fig 1:**
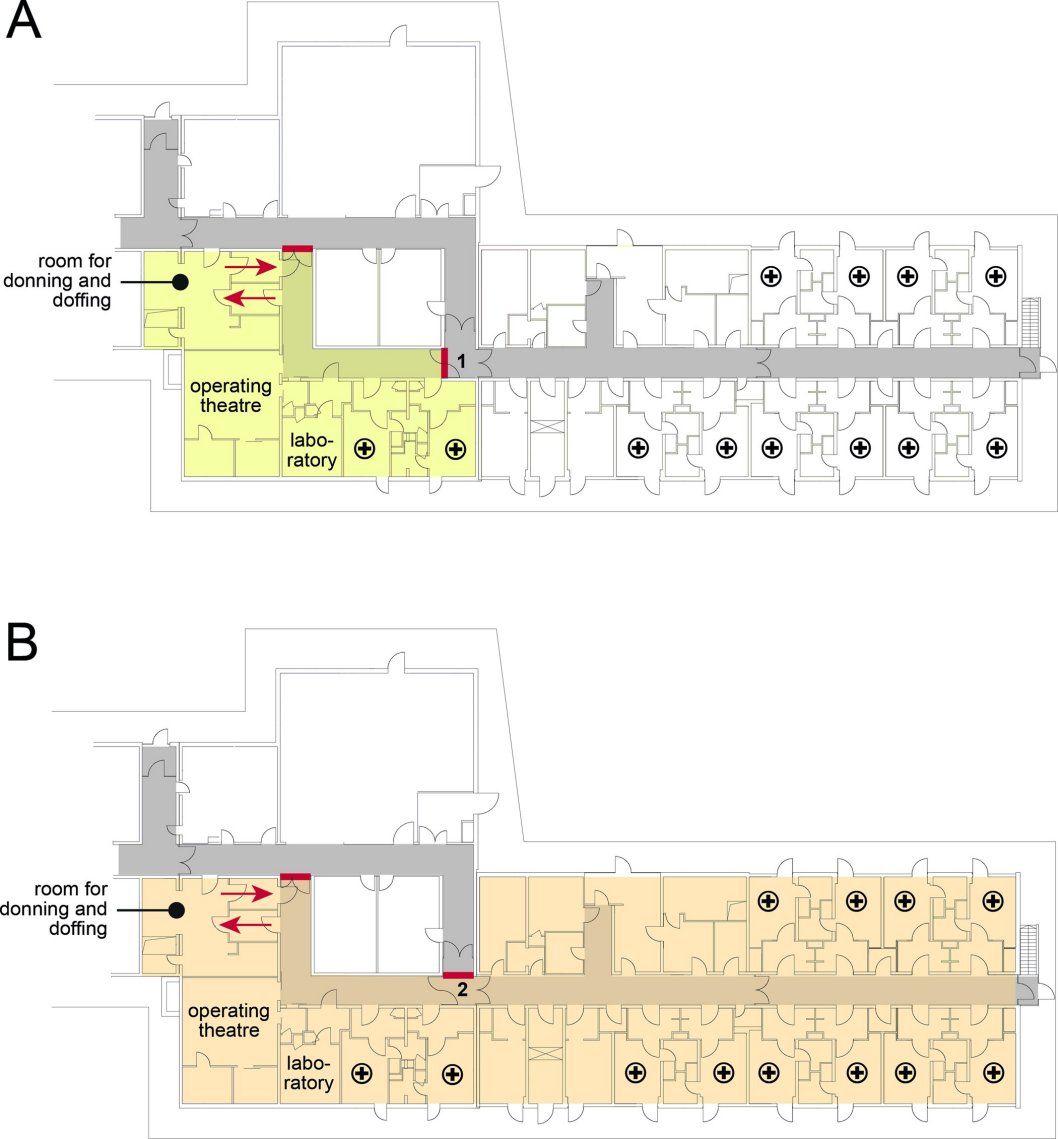
Plan of the high-level isolation unit (HLIU). **A** small version with two patient rooms, **B** large version with twelve patient rooms. Red bars denote doors, which seal the isolation area (marked by light colors) from other areas, arrows denote entry and exit of the isolation area, **+** denotes patient room with anteroom. In the small and large version of the HLIU, door (1) and (2) are closed, respectively (see **A** and **B**).

The Department for Infectious Diseases and Respiratory Medicine at Charité–Universitätsmedizin Berlin runs the Berlin HLIU. As of November 2021, this department employs 148 nurses and 74 medical doctors on seven wards on the three different campuses of Charité—Universitätsmedizin Berlin, among them one medical intensive care unit (ICU) with 18 in-patient beds providing high level care for patients with acute respiratory distress syndrome (ARDS). The HLIU and the ICU are located on different campuses. The HLIU is Germany’s largest with a maximum capacity for 20 patients. In daily routine, the ward provides in-patient care with a special focus on infectious or respiratory diseases, however, one room is constantly reserved for potential patients with HCID. The HLIU can be operated in two different ways depending on the number of HCID in-patients, by differential activation of negative pressure: as a HLIU with two rooms and as a large HLIU with 12 rooms with a maximum capacity for four and 20 in-patients, respectively (Fig 1).

### Alerting

In case of admission of a patient with HCID, staff is alerted by a digital server for alerting and communication (DAKS). Three different levels of alerting are determined. Level I is activated if a single patient is admitted in stable condition. Alerting concerns doctors and nurses from the HLIU and all relevant institutions within Charité–Universitätsmedizin Berlin (i.e. managing board, crisis management group, department for infection prevention and hygiene, administration and maintenance, logistics, security). The intensive care team is informed but intensive care staff may keep on with their usual activities. Level II is activated if an unstable patient is admitted. In addition to the aforementioned persons and institutions, the intensive care team is alerted. The intensive care team then splits in two groups. One group takes over the medical management of the unstable patient with HCID, the other group ensures ongoing treatment of patients on the ICU, where usually >90% of in-patient beds are occupied. As this division critically reduces the team on the ICU, all remaining ICUs of this campus of Charité–Universitätsmedizin Berlin delegate one staff member to reinforce the ICU-team. Level III is meant for catastrophic events with large numbers of patients with potential HCID. In this case all ICUs are alerted. Announcements of the DAKS for all three levels are prespecified. Additionally, there is an option for selected staff to deliver free text messages via the DAKS. Activation of the HLIU is expected to be completed within three hours. The process of putting into operation is complex as many materials, which are used in daily routine must be removed from the isolation area and exchanged by material for HCID patient care. Regularly updated packing lists with all necessary material ensure a time efficient process. The technical preparation of the HLIU (e.g. establishment of negative pressure in four different levels) can be realized within one hour. Technical details are beyond the scope of this article.

### Team

The HLIU team is led by ten staff members: two nurses who routinely work in the HLIU, four intensive care nurses, and four consultant physicians with specialization in infectious diseases (3), and intensive care medicine (1), respectively. All team leaders meet at least quarterly, more frequent meetings are scheduled depending on needs. The HLIU team consists currently of 32 nurses and 34 medical doctors trained on a regular basis. Recruitment of new staff is constantly necessary due to staff turnover. All medical staff who are not yet part of the HLIU team are contacted yearly and asked for their interest to become team members. Interested staff undergo a one-day basic training session before they may integrate in the HLIU team. Staff from other departments are welcome to the team, however, other departments are not yet regularly targeted for staff recruitment as the Department for Infectious Diseases and Respiratory Medicine has a sufficient staff pool and planning of training is more convenient if organized in a single department.

#### Standard operating procedures (SOP)

Numerous SOP have been developed specifically for the HLIU (a list of current SOP is available in the supplementary material). SOP are updated regularly and new SOP are developed according to needs (team leaders decide on the need after feedback from team members).

#### Training

Until 2016, nurses and doctors practiced independently in half-day monthly training sessions with focus on donning and doffing PPE. After the large EVD outbreak in West Africa in 2014–2015 evidence-based guidelines for supportive care were developed [12]. It was perceived that the focus should shift from mere isolation of patients to complex medical management of patients in critical condition, as well as focus on training with an interprofessional team approach: nursing staff and doctors, infectious disease specialists and intensive care specialists. Therefore, team leaders convened to design training sessions as follows.

Full day training sessions now take place monthly. Participants receive relevant documents one week prior to the training sessions so they have time to prepare for the training. Two to three team leaders consisting of at least one nurse and one physician supervise eight to ten participants. Attendees record their participation by signature. Doctors and nurses exercise together reflecting real life practice. Training sessions begin and end with a questionnaire, which addresses different aspects of preparedness and competence (e.g. “I feel competent with airway management in the HLIU”). The same questions are asked before and after training on the same sheet enabling assessment of the training effect. Participants are asked to respond to the listed statements on a 5-point Likert scale (with extremes labelled as “I do not agree at all” at the lower and “I fully agree” at the higher end). A perception survey complements the pre-training questionnaire. It assesses general attitudes to training necessity and training concepts (e.g. “Training on a regular basis is necessary to prepare for managing patients with HCID”). Participants are asked to respond to the listed statements on a 5-point Likert scale (with extremes labelled as “I do not agree at all” at the lower and “I fully agree” at the higher end). Participation in the questionnaire and the perception survey is anonymous. Prior to the practical session one of the trainers lectures on a relevant HLIU topic (e.g. guidelines from the German Committee for Biological Agents in the workspace (ABAS), SOP for sample taking and shipping, management of patients with suspicion of viral hemorrhagic fever). Participants are then shortly guided through the HLIU. This is deemed necessary to re-familiarize participants with the location of materials within the HLIU. Participants can optionally take their weight before donning and after doffing PPE (in order to assess weight loss due to perspiration in the PPE). Next, participants don PPE (we use a fully encapsulating lightweight suit that protects against liquid chemicals and solid particulates (EN type 3/4/5) as well as infective agents (EN type 3B/4B/5B) in combination with a powered air purifying respirator with combination filters against gases and particles (PM Chemical Grey, PM Atemschutz, Germany)). The process of donning PPE is done in pairs, i.e. two colleagues mutually verify that all precautions of a given checklist are met. Depending on the level of experience this process may be supervised by the trainers. Participants then enter the negative pressure area in full PPE. They have to fulfil tasks, which are adapted to real life scenarios. Attendees are advised to focus on good organization of the workplace and hygiene. Rescue of unconscious colleagues is always simulated in the training sessions as this is regarded as crucial for staff safety. The full range and timing of training contents can be seen in Table 1. Attendees spend three hours in full PPE, which corresponds to a real shift. Further topics after decontamination and doffing PPE include exercises in light PPE (i.e. without powered respirator), feedback, and the second part of the questionnaire.

**Table 1:** Timetable of training sessions.

Activity	Remark	Approximate length of time (min)^a^
Preparation of training session	Documents are provided for attendees one week prior to training	NA
Questionnaire regarding preparedness and competence	At beginning and end of training	10 each
Perception survey		5
Lecture	Different topics with relevance for HLIU (see text)	30
Guided tour through HLIU	Demonstration of location of materials and devices	10
Donning PPE		30
Execution of checklist prior to entrance of negative pressure area	Checklist addresses safety issues (e.g. PPE is errorless) and personal issues (attendee healthy and ready to enter HLIU)	15
Airway management	Oxygen administration, sedation, intubation (laryngoscope and video laryngoscope), suction on airway trainer	45
Venous catheter management	Insertion of central line and peripheral line on patient simulator	45
Chest tube management	Insertion of chest tube on patient simulator	45
Nasogastric tube management	Insertion of nasogastric tube on patient simulator	10
Urinary catheter management	Insertion of urinary catheter on patient simulator	10
Organization of workplace	Communication in PPE, spatial management, waste management, cleaning	NA
Sample investigation	Blood count, blood chemistry, coagulation test, blood gas analysis, rapid diagnostic test for malaria on point-of-care testing devices; microscopy on video microscope	45
Sample processing	Packing and labelling of samples under directive UN 2814 prior to posting for reference laboratory for specific diagnostics	15
Staff rescue	Emergency decontamination and doffing of staff with simulated syncope	15
PPE problems	Low respirator battery, hole in PPE, needle stick injury	10
Decontamination and doffing PPE		10
Exercises in light PPE (i.e. without powered respirator)	Management of a patient with low suspicion of HCID	45
Joint clearance of premises		20
Feedback		15

*NA* not applicable, *PPE* personal protective equipment, *HLIU* high-level isolation unit, *HCID* high-consequence infectious disease.

^a^Activities may take place simultaneously.

#### Exercises

Exercises take place regularly once a year. They follow different scenarios, and numerous partner institutions can be involved depending on the given scenario. Preparation is complex. Three exercises were conducted during the last three years with different foci. Post processing is facilitated by one of the team leaders. Participants, referees, and observers discuss perceived shortcomings and set up action plans to eliminate deficits.

### First exercise

An extensive exercise was carried out in October 2017 involving seven institutions (Criminal Investigation Department Berlin Police, Federal Criminal Police Office, German Federal Police, RKI, Berlin Senate of Health, Berlin Fire Brigade, and Charité–Universitätsmedizin Berlin). The exercise scenario included raid of a bioterrorism laboratory with three suspects by Special Forces. One suspect was found dead, and one suffered from a gunshot injury to his right thigh but also showed respiratory symptoms. The living subject (who was initially played by a volunteer) was admitted to the HLIU with security patrols. After the first body check and transfer to a patient bed the volunteer was replaced by a patient simulator (HAL®S3000, Gaumard, USA). Exercise tasks included intensive care management (airway management, catheter management, volume resuscitation, transfusion of blood products), orthopedic surgery (external fixation of a dislocated fracture), and sample investigation and sample processing. The corpse of the other subject was simulated by a real corpse provided by the Institute for Forensic Medicine with authorization from the Public Prosecution Authority. This corpse was brought to the HLIU in a second transport vehicle. Experts from the Institute for Forensic Medicine performed a full body forensic autopsy in full PPE according to the German code of criminal procedures §§87–89 [13] and according to the Guidelines of the Association of the Scientific Medical Societies in Germany (AWMF) [14]. The German code of criminal procedures stipulates that a forensic autopsy has to be performed by two medical doctors including one board certified expert in legal medicine. Medical doctors were supported by an autopsy technician. Also, a team of two members of the forensic technical department (trace evidence and dactyloscopy), a professional photographer, and a criminal investigator of the Berlin Police were involved in the postmortem forensic procedures. As the teams from the Institute for Forensic Medicine and the Berlin Police were only partially familiar with the provided PPE and the premises of the HLIU they were closely supported and supervised by HLIU staff. In addition to the complete forensic autopsy samples and findings for identification (DNA-material, fingerprints, and dental status) and samples for toxicological analysis were preserved. Furthermore, blood and respiratory samples from both the patient and the corpse were delivered to the RKI. Two referees observed the whole exercise. One of them was an external expert designated by the STAKOB, the other one was a team leader from our HLIU who was unaware of the exercise scenario.

### Second exercise

The second exercise in February 2018 focused on internal workflow. Therefore, no partner institutions were involved. The exercise spanned 24 hours with the majority of activities taking place during the night. Within the scenario, a voluntary performer presented to the emergency department stating to have shortly returned from Uganda where she had been working in a medical project. Signs and symptoms were compatible with a HCID. The scenario included medical tasks (management of an initially stable patient with rapid clinical deterioration and subsequent death), laboratory tasks (blood gas analysis, chemistry, blood count, coagulation assay, rapid diagnostic testing (malaria, dengue), blood film microscopy with video microscope, blood culture and pathogen identification, blood typing), organizational tasks (preparation of HLIU, patient transport, shift handover, handling of a dead body), and emergency tasks (rescue of unconscious colleague in PPE, needle-stick injury, tear in PPE). Contaminated waste was autoclaved (Matachana EC 2120, Barcelona, Spain). Function of the autoclave was assessed by insertion of nineteen bio indicators (*Geobacillus stearothermophilus*) in different positions within the waste. Two team members were exclusively in charge of observing the exercise.

### Third exercise

The third exercise in August 2019 comprised again several institutions (Airport Berlin Brandenburg, German Federal Police, Berlin Police Department, Berlin Fire Brigade, Berlin Public Health Service, and Charité–Universitätsmedizin Berlin). Important foci of this exercise were the interaction and collaboration between these institutions and again the workflow on the HLIU. Within the setting, a patient, who was played by a volunteer, presented in the airport with reduced level of consciousness and bleeding from the nose. Anamnesis revealed recent travel to Sierra Leone. A suspicion of viral hemorrhagic fever was raised and the patient was transferred to the HLIU. After admission, a patient simulator replaced the volunteer. The scenario for this patient was similar to the second exercise with management of a complex medical case over several three-hour night shifts. In addition, a symptomatic contact of the first patient, again played by a volunteer, presented to the emergency department of Charité—Universitätsmedizin Berlin. This patient was transported to a second room within the HLIU and managed by a different team. As evaluation of the preceding exercises had revealed major communication problems, one important focus of this exercise was testing of a new communication system (Riedel Bolero, Riedel, Germany). Testing was facilitated by technicians from the company. Two team members were exclusively in charge of observing the exercise.

#### Statistics

Results from the questionnaire and the perception survey are shown as medians and interquartile ranges (IQRs) of points given by participants on the 5-point Likert scale. To assess the immediate impact of training, median results before and after training were compared with the Wilcoxon signed-rank test. The long-term training impact was evaluated by assessing the correlation of median results before training (dependent variable) with the number of prior trainings (independent variable) with the Spearman’s rank correlation. Two-tailed p values of less than 0.05 were considered to indicate statistical significance. Data analysis was performed using SPSS (IBM, USA), version 27.

#### Ethical considerations

We did not seek approval from the ethics committee for this study for the following reasons. This study does not involve any patient data. Participation in the HLIU team is voluntary. Participants actively express their wish to become team members. During training sessions, a questionnaire and a perception survey are handed to the trainees, which address aspects of preparedness and attitudes, respectively. Participants are verbally informed that filling of these documents serve to assess the impact and relevance of training, that participation is voluntary, and that participation–if the participant is inclined to participate–is anonymous. Training participants choose to fill or not to fill the documents. Consent is not formally recorded. No data can be traced back to the participants (see supplementary material for the original documents). No other personal data are collected in this study.

## Results

### Training

Training sessions were held monthly from May 2017 up to now except for the first wave of the SARS-CoV-2 pandemic in Germany, when training sessions were suspended in April and May 2020 and resumed in June 2020, and the second larger wave, when training sessions had to be suspended for seven consecutive months from November 2020 to May 2021.

#### Questionnaire

**Fig 2:**
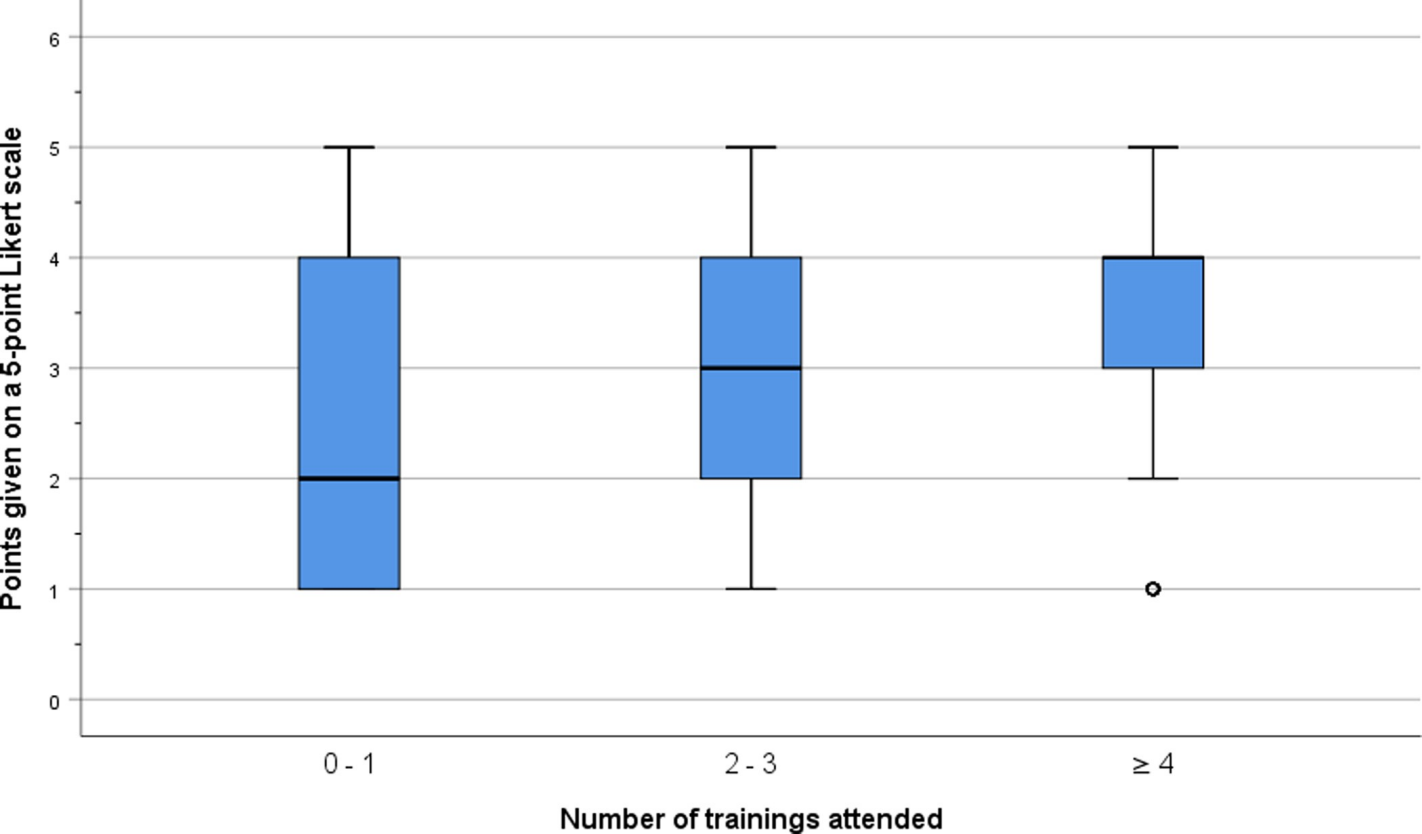
Perceived competence depending on the number of trainings attended. Box plot of points on the 5-point Likert scale (with extremes labelled as “I do not agree at all” at the lower and “I fully agree” at the higher end). The lower bound of the box indicates the first quartile, the thick line indicates the median, and the upper bound of the box indicates the third quartile. The lower and upper whisker indicate minimum and maximum values, respectively. The circle represents outliers (i.e. < first quartile– 1.5*IQR). 0–1, 2–3, and ≥ 4 trainings had been attended by 43, 40, and 33 participants, respectively. Results show overall agreement with 13 questions addressing competence (e.g. “I feel competent with airway management in the HLIU”) from participants who had so far participated in 0–1, 2–3, and ≥ 4 training sessions.

116 questionnaires were filled by 58 doctors and 58 nurses. Considering the current team of 32 nurses and 34 medical doctors, this means that each participant filled an average of 1.8 questionnaires. When filling the questionnaire, participants had attended a median of 2 training sessions (IQR 1–4). 0–1, 2–3, and ≥ 4 trainings had been attended by 43, 40, and 33 participants, respectively. Agreement with the listed statements was significantly higher for all but one statement after training compared to before training indicating a positive effect of training sessions on competence. The only statement where no effect of training could be detected referred to the patient data management system (Table 2). The correlation between the number of training sessions and overall agreement with the listed statements was assessed by comparing overall agreement with the listed statements before training from participants who had so far participated in 0–1, 2–3, and ≥4 training sessions, respectively. The correlation was significant (*r*_*s*_ = .243, *p* < .01, *n* = 1,481). Perceived competence dependent on the number of trainings attended is displayed in Fig 2.

**Table 2:** Perception of competence before and after training.

	before training	after training	*p* ^*a*^
I feel well prepared for treating patients with HCID	3 (2–4)	4 (3–4)	<0.001
Our department is well prepared for the treatment of patients with HCID	4 (3–4)	4 (4–5)	<0.001
I feel competent with donning PPE	3 (2–4)	4 (4–5)	<0.001
I feel competent with decontamination and doffing PPE	3 (2–4)	4 (3–4)	<0.001
I know how to act in case of an empty blower-battery	3 (2–4)	4 (4–5)	<0.001
I know how to act in case of a pinprick injury with risk of infection	3 (2–4)	4 (3–5)	<0.001
I feel competent with staff rescue within the HLIU	3 (2–4)	4 (3–5)	<0.001
I am confident that I will be rescued competently if I suffered a medical emergency while wearing PPE	4 (3–4)	4 (4–5)	<0.001
I feel competent with laboratory diagnostics within the HLIU	2 (1–3)	3 (2–4)	<0.001
I feel competent with placing a central line within the HLIU	3 (1–3.5)	4 (3–4)	<0.001
I feel competent with airway management within the HLIU	3 (2–4)	4 3–4)	<0.001
I feel confident with the patient data management system	4 (3–5)	4 (3–5)	0.098
Communication via the communication system within the HLIU works well	3 (2–3)	3 (2–4)	<0.001
**Overall**	**3 (2–4)**	**4 (3–5)**	**<0.001**

*HCID* high-consequence infectious disease, *PPE* personal protective equipment, *HLIU* high-level isolation unit.

^a^Determined by Wilcoxon signed-rank test. Data show median scores (IQR) on a 5-point Likert scale (with extremes labelled as “I do not agree at all” at the lower and “I fully agree” at the higher end).

#### Perception survey

29 nurses and 32 physicians filled the perception survey. Participants rated joint trainings for nurses and doctors at regular intervals as highly important. Median scores were 5 on the 5-point Likert scale, thus indicating maximum agreement (IQR 5–5). Similarly, participants perceived a global increase in HCID with a high likelihood that patients with HCID could be treated in our unit (median score 4, IQR 3–5, see supplementary material).

#### Exercises

Comments from referees and observers and feedback from participants revealed numerous issues, where improvement was deemed necessary. Lack of organization of the workplace and impaired communication with team members in the negative pressure area were considered as important shortcomings in the first and the second exercise. All external specialists who had been unfamiliar with the HLIU and who had to work in the isolation area to fulfil specific tasks (e.g. orthopedic surgeon carrying out external fixation of a dislocated fracture, forensic pathologists performing full body forensic autopsy, microbiologist performing pathogen identification) expressed they could work well and safely under the supervision of the HLIU team. A comprehensive list of issues raised during post processing of all three exercises with following consequences and status quo can be seen in Table 3.

**Table 3:** Post processing of exercises and further implications.

Deficits identified during post processing of exercises	Consequences and status quo (as of January 2022)
Inflexible automatic alerting of all staff irrespective of patient status	Allocation of different levels of alerting according to number of patients and patient status
	Automatic alarm now in place with three different stages of alerting according to number of patients and patient status (see text)
Communication systems between HLIU and external institutions not compatible	Testing and procurement of a new communication system (see text for detailed information)
	Constant video-observation of isolation area by one designated staff member
Temporary blackout of communication system when too many participants	
	Installation of alternative means of communication (internet based communication via Skype for business, Microsoft®, USA)
Insufficient separation of communication channels between different groups in isolation area	
Insufficient organization of the workplace (e.g. gloves and sharp safes not accessible at all times, hand disinfection not always done, insufficient cleaning of floor, insufficient waste management)	Focus on organization of the workplace in training sessions and exercises: trainees are reminded that no extra staff is available for cleaning and disinfection and that the indications for hand hygiene remain the same when wearing full PPE (i.e. the outer pair of gloves must be discarded after each patient contact, the inner pair of gloves, which are firmly connected to the suit, are then disinfected and ultimately, a new pair of outer gloves is put on)
Materials missing in the isolation area must be brought to the isolation area by extra staff after donning PPE	Construction of material air lock: process ongoing, prerequisites for procurement and installation are non-powered, mechanical opening, easy maintenance, and no interference with the ventilation system of the HLIU
Staff not familiar with HLIU has difficulty in coping with PPE and premises	Allocation of staff for supervision of external personnel
Some staff members have too many tasks that cannot be performed simultaneously	Definition and description of specific responsibilities
	Staff is allocated to specific responsibilities
Difficult handling of symptomatic contact as existing SOP is deemed insufficient	SOP for treatment of stable patients with low suspicion of HCID updated
	Management of stable patients with low suspicion of HCID is integrated in training sessions (exercise in light PPE (i.e. without powered respirator))
Activation of HLIU time consuming	Clear labeling of drawers and shelves (e.g. with photos)
	Creation of additional storage area

*HLIU* high-level isolation unit, *PPE* personal protective equipment, *SOP* standard operating procedure

### First exercise

The bioterrorism exercise was evaluated extensively and jointly by the participating institutions. One major outcome of this joint evaluation was a handbook for the management of locations, which are contaminated by biological hazardous material [15]. The handbook gives recommendations on possible actions for police and public health experts in bio terroristic attacks. Contents of the handbook are subject to confidentiality.

### Second exercise

Retrieval of the bio indicators within the autoclaved waste was difficult. Five out of nineteen bio indicators could be located and could be excised for analysis with cutting tools. All of them were sterile indicating proper functioning of the autoclave. The remaining fourteen bio indicators could not be spotted within the waste. This was attributed to the transformation of the waste, which was highly condensed by autoclaving. The autoclave undergoes routine checking by our facility management. No problems have been identified in recent years.

### Third exercise

Testing of the new communication system was unique to the third exercise. It was unanimously perceived as an important advance in both safety and management capabilities. Communication groups with the old communication system had to be pre-set. Switching to other conference calls was not possible. There was no option for “push-to-talk” communication (i.e. every sound was spread to all participants in the conference call resulting in a high noise level). Battery life was short (i.e. sometimes the battery did not last for the duration of a three-hour shift) and voice quality was poor. The new communication system is based on advanced digital enhanced cordless telephone technology (DECT) enabling higher intelligibility. Wireless receivers feature six intercom channels and a separate “reply” button allows flexible use of conference calls and point-to-point as well as push-to-talk communication. The receiver is worn inside the PPE. Buttons are large enough to be operated through the PPE. Battery life is longer than the duration of shifts.

In addition, booting of the HLIU was time consuming mainly due to a prolonged process of removing unneeded materials and the delivery and shelving of necessary materials (Table 3).

#### Impact of the SARS-CoV-2 pandemic

The SARS-COV-2 pandemic interfered with activities of the HLIU. At the height of the pandemic on January 6, 2021, 240 coronavirus disease (COVID-19) patients were admitted to Charité—Universitätsmedizin Berlin. Out of these, 153 were treated on designated ICUs, 114 were mechanically ventilated and 37 received extracorporeal membrane oxygenation (ECMO). Both the medical ICU of the Department for Infectious Diseases and Respiratory Medicine and the HLIU became designated solely for care of patients with COVID-19. Trainings had to be suspended for several months (see above). Team sessions were shortened and held as video-conferences. The yearly exercise, which had been planned for 2020 had to be postponed. Training sessions resumed in June 2021, when 7-day incidence rates for SARS-CoV-2-infections in Berlin had reduced substantially (33/100,000 as compared to a maximum of 224/100,000 on 22^nd^ of November, 2020).

On a national level, STAKOB closely followed the evolution of the epidemic from the beginning. When the first cases of presumed viral pneumonia were reported from Wuhan, China in December 2019, a novel pathogen with unknown properties seemed likely and weekly STAKOB telephone-conferences were organized. Thus, cooperation of national experts from public health and the German HLIU was strengthened.

## Discussion

Working in a HLIU can be challenging in several ways. Wearing full protection PPE is physically strenuous. Vision, sense of hearing, sense of touch, olfaction, and flexibility of movements are greatly impaired. Verbal communication is enabled by technical means, which demand high discipline in communication. Medical tasks, which are performed routinely in everyday settings (e.g. insertion of a central line) may become difficult when wearing PPE in a HLIU. Psychological stress may arise from fear of exposure to potentially deadly pathogens and the unfamiliar working environment. Furthermore, many scenarios (e.g., necessity of surgery, childbirth, necessity for autopsy) require external staff, who need to be supervised closely.

No patient with HCID has been treated in the HLIU of Charité–Universitätsmedizin Berlin during the last decade. One patient was airlifted to our HLIU during the large EVD epidemic in West Africa in 2014–2015. However, tests for EVD turned out to be negative. There are reports from other German HLIU, where both EVD [16, 17] and Lassa fever [18] patients were treated in recent years. These experiences were shared in detail in STAKOB meetings. Only two out of nine HCID patients survived, who had been treated with different HCID (five cases with Lassa Fever, one case each with CCHF, Yellow Fever, MVD, and SARS) in Europe between 1997 and 2008 [2]. Contrarily, a larger observational retrospective study by Uyeki et al. shows that patients with viral hemorrhagic fever (EVD in particular) may have a good chance of survival (>80%) if treated with close monitoring and aggressive supportive care in Europe and the United States [5]. The availability of specific drugs (like ansuvimab-zykl or the combination of atoltivimab, maftivimab and odesivimab in the case of EVD) may further reduce case fatality rates. The case fatality rate during the EVD outbreak in West Africa was initially >70%. Individualized clinical supportive care including symptom control, laboratory-facilitated diagnosis of organ dysfunction, treatment of shock with fluids and electrolytes, and rapid diagnosis or empirical treatment of concomitant illnesses decreased the case fatality rate to approximately 40% [12]. Thus, a HLIU should nowadays not be a place where patients are merely isolated accepting poor survival but provide all aspects of modern intensive care aiming for best possible patients’ outcomes. We therefore deemed necessary to focus on different aspects of supportive care that may be particularly challenging in a HLIU.

Considering the aforementioned aspects, our approach includes several strategies: (1) training must take place at regular intervals within mixed teams of doctors and nurses, (2) training should focus on an interprofessional approach including intensive care, (3) current SOP should cover relevant tasks within the HLIU, (4) extensive exercises are necessary to simulate real life scenarios and train the collaboration with different institutions. Participants judged the possibility of a HCID patient requiring treatment in our department to be high and saw the need for regular training sessions in mixed teams of doctors and nurses. We could show that the perceived competence for specific skills increased through training sessions, as indicated by higher scores on the Likert scale for all but one statement (the only statement where no significant effect of training could be detected referred to the patient data management system where competence was already high before training). Furthermore, there may be a correlation between the number of training sessions attended and the perception of competence. This suggests that our training sessions were effective to some extent. There is, however, room for further improvement. This is partly reflected in the fact that statements regarding skills within the HLIU did not achieve maximum agreement on the Likert scale and, surely, our questionnaire did not reflect the whole breadth of the management of a HCID patient. One major remaining challenge is staff turnover, which affects all teams who work together over several years at least to some extent. Herstein et al. administered an electronic survey to 56 HLIU in the United States. They found that significant numbers of staff were recruited and trained with little guidance [19]. Continuous efforts in recruitment and teaching are therefore necessary.

The complexity of the treatment of a patient with HCID within our HLIU was addressed with yearly exercises. These exercises were meant to simulate different real-life scenarios involving numerous partner institutions. Evaluation of such exercises is challenging as settings differ greatly and no single approach can adequately capture all aspects of the different scenarios. To have a good overview, at least one team member was designated solely for observation. In addition, all participants were asked to give feedback, and one exercise was attended by an external referee. Findings were discussed in team sessions and action plans were generated, which were then processed step by step. Several shortcomings could thus be eliminated or at least alleviated, among others, the difficulty in communication and the handling of external staff within the HLIU. There was a strong feeling within the team that exercises–although planning and post processing is time-consuming–are of the utmost importance for the preparation of admitting patients with HCID.

Evidently, external staff may perceive working in a HLIU differently. We got feedback from experts from different institutions, with whom we had been working during the exercises. All of them expressed that they had been supervised well and that they could fulfil their specific tasks. We believe the same may be true for experts from other specialties like obstetrics and pediatric care who were not involved in our exercises. For medico legal experts the use of normal autopsy facilities is untenable in corpses with suspected HCID. Even though it was possible to fulfil the legal regulations for a forensic autopsy in PPE within the first exercise, further training of these teams and enforcement of facilities for HCID are necessary. We have conducted one training session in the Institute for Forensic Medicine subsequent to our exercise. However, again a continuous effort is needed to sustainably ensure adequate skills.

The SARS-CoV-2 pandemic interfered substantially with our activities. The Department for Infectious Diseases and Respiratory Medicine, which runs the HLIU, was disproportionately affected by the pandemic as COVID-19 is an infectious disease that mainly affects the lungs.

On the national level, the capacity to admit and treat HCID patients was limited at the height of the pandemic. However, this limitation was not regarded as critical as, in the first place, imported cases were unlikely due to the breakdown of international travel, and, in the second place, the close collaboration between German treatment centres would have enabled a flexible allocation of patients, i.e. patients would have been allocated to the treatment centre with the least workload due to COVID-19 with extra support from other treatment centres. On the local level of our HLIU, COVID-19 patients from the HLIU could have been transferred to other wards within Charité—Universitätsmedizin Berlin or to other hospitals in Berlin in order to have the entire HLIU for the treatment of patients with HCID. Furthermore, all ICUs from Charité—Universitätsmedizin Berlin could have delegated staff to our ICU so that our team members would have had the capacity to work on the HLIU. The expertise of the HLIU in dealing with infections with unknown properties was appreciated. This was reflected in the fact that many of the first patients with SARS-CoV-2 infection in Germany were treated in centres with HLIU. Thus, HLIU may not only serve as treatment centres for patients with HCID but also as centres for patients with infections due to novel pathogens.

Our study has several limitations. First, this is the description of the approach of a single centre, which cannot be easily adopted by other centres. Physical structures, technical facilities, and team composition vary greatly between different countries. Even within Germany, with the STAKOB as common coordinating institution, it was felt that each centre should define necessary prerequisites for patient admission independently, as situations in the respective centres were too different for a common approach. Though, we believe that our work may give some ideas about what aspects should be considered when preparing a unit for the admission of patients with HCID. The different aspects described in this article may then be adapted to the local situations. Second, our approach is the result of intense discussions among team members and not based on evidence. We do not know whether different foci would have led to a better preparation. However, every training was followed by feedback and exercises were thoroughly evaluated. Thus, shortcomings, which were identified during evaluation, could directly be addressed. Third, although we tried to evaluate the impact of training by assessing skills with a questionnaire before and after training, we only addressed perceived competence, which does not necessarily translate into actual competence. However, many of the staff members regularly work in the ICU and may adequately perceive how well they can perform routine ICU tasks (e.g. insertion of a central line) when wearing full PPE. Fourth, we could never test whether our preparation withstands the challenges of the treatment of a real patient with HCID as no patient with HCID was treated in Berlin during the last decade. We had on several occasions signaled our readiness for admissions but allocation of patients was beyond our influence. Fifth, we are aware that psychosocial support is crucial for patients with HCID as well as their families. So far we have not put effort into training the team members in this regard. However, all staff members have experience in supporting patients psychologically in critical situations and during the SARS-CoV-2 pandemic, we collaborated with the division of psychosomatic medicine of the Charité—Universitätsmedizin Berlin. Patients received regular visits by the psychosocial team and additionally, a tablet computer was used for virtual psychosocial support.

Preparation should be regarded as a flexible continuum. Future challenges may arise from changing distribution ranges of pathogens and intensified human contact with ecosystems. These may lead to infections with previously unknown pathogens and more outbreaks due to known HCID pathogens. We believe HLIU are necessary for future responses. This article describes some issues that may be of interest for multidisciplinary teams training for infrequent but high consequence events.

## Supporting information

S1 File List of current standard operating procedures. (DOCX)

S2 File Perception survey. (DOCX)

S1 Questionnaire (DOCX)

S1 Data Raw data questionnaire and perception survey. (XLSX)
