# Supplementary material for: Preparing for patients with high-consequence infectious diseases: Example of a high-level isolation unit
Source: PLoS One. 2022 Mar 3;17(3):e0264644. doi: 10.1371/journal.pone.0264644 (PMC8893674; doi:10.1371/journal.pone.0264644)
Supplement: S1 File — (DOCX) [file pone.0264644.s001.docx]

# Standard operation procedures (SOPs) of the High-level isolation unit (HLIU) at Charité Universitätsmedizin Berlin

## Donning and doffing

- Donning and doffing personal protective equipment (PPE)
- Donning and doffing PPE for decontamination assistant

## Decontamination

- Decontamination of PPE after leaving the HLIU
- Processing of reusable contaminated materials
- Disposal of contaminated waste

## Procedures

- Completing checklist after donning PPE before entering the HLIU
- Self-monitoring when wearing PPE within the HLIU
- Transport of a patient with suspected HCID from the emergency department to the HLIU
- Rescue of unconscious colleagues wearing PPE
- Pinprick injury with risk of infection
- Ruling out the suspicion of a high-consequence infectious disease (HCID) in patients with low risk for HCID
- Management of patients considered at high risk for HCID
- Airway management within the HLIU
- Central line placement within the HLIU
- Handling of a corpse with HCID
- Processing of biological samples including shipment to reference laboratory
- Inward transfer of materials

## Technical issues

- Rules for communication within the HLIU
