# Supplementary material for: Preparing for patients with high-consequence infectious diseases: Example of a high-level isolation unit
Source: PLoS One. 2022 Mar 3;17(3):e0264644. doi: 10.1371/journal.pone.0264644 (PMC8893674; doi:10.1371/journal.pone.0264644)
Supplement: S2 File — (DOCX) [file pone.0264644.s002.docx]

Date:

Professional category: doctor  nurse

# Perception Survey

Please tick on the scale from 1 to 5.

1, « I disagree completely » ; 5, « I fully agree ».

|  | 1 | 2 | 3 | 4 | 5 |
| --- | --- | --- | --- | --- | --- |
| 1. The threat of diseases caused by highly pathogenic pathogens is increasing worldwide |  |  |  |  |  |
| 1. There is a realistic possibility that patients with HCIDs will be admitted to our HLIU |  |  |  |  |  |
| 1. Training on a regular basis is necessary to prepare for managing patients with HCIDs |  |  |  |  |  |
| 1. Training sessions should preferably be carried out jointly for doctors and nurses |  |  |  |  |  |
| 1. I can easily participate in the training sessions in the context of my professional obligations |  |  |  |  |  |
