# Supplementary material for: Preparing for patients with high-consequence infectious diseases: Example of a high-level isolation unit
Source: PLoS One. 2022 Mar 3;17(3):e0264644. doi: 10.1371/journal.pone.0264644 (PMC8893674; doi:10.1371/journal.pone.0264644)
Supplement: S1 Questionnaire — (DOCX) [file pone.0264644.s003.docx]

Date:

Professional category: doctor  nurse

Number of training sessions attended so far (not including the current session) ______

Please tick on the scale from 1 to 5.

1, « I disagree completely »; 5, « I fully agree ».

|  | before training | | | | |  | after training | | | | |
| --- | --- | --- | --- | --- | --- | --- | --- | --- | --- | --- | --- |
|  | 1 | 2 | 3 | 4 | 5 |  | 1 | 2 | 3 | 4 | 5 |
| 1. I feel well prepared for treating patients with HCIDs | 🞎 | 🞎 | 🞎 | 🞎 | 🞎 |  | 🞎 | 🞎 | 🞎 | 🞎 | 🞎 |
| 2. Our department is well prepared for the treatment of patients with HCIDs | 🞎 | 🞎 | 🞎 | 🞎 | 🞎 |  | 🞎 | 🞎 | 🞎 | 🞎 | 🞎 |
| 3. I feel competent with donning PPE | 🞎 | 🞎 | 🞎 | 🞎 | 🞎 |  | 🞎 | 🞎 | 🞎 | 🞎 | 🞎 |
| 4. I feel competent with decontamination and doffing PPE | 🞎 | 🞎 | 🞎 | 🞎 | 🞎 |  | 🞎 | 🞎 | 🞎 | 🞎 | 🞎 |
| 5. I know how to act in case of an empty blower-battery | 🞎 | 🞎 | 🞎 | 🞎 | 🞎 |  | 🞎 | 🞎 | 🞎 | 🞎 | 🞎 |
| 6. I know how to act in case of a pinprick injury with risk of infection | 🞎 | 🞎 | 🞎 | 🞎 | 🞎 |  | 🞎 | 🞎 | 🞎 | 🞎 | 🞎 |
| 7. I feel competent with staff rescue within the HLIU | 🞎 | 🞎 | 🞎 | 🞎 | 🞎 |  | 🞎 | 🞎 | 🞎 | 🞎 | 🞎 |

|  | before training | | | | |  | after training | | | | |
| --- | --- | --- | --- | --- | --- | --- | --- | --- | --- | --- | --- |
|  | 1 | 2 | 3 | 4 | 5 |  | 1 | 2 | 3 | 4 | 5 |
| 8. I am confident that I will be rescued competently if I suffered a medical emergency while wearing PPE | 🞎 | 🞎 | 🞎 | 🞎 | 🞎 |  | 🞎 | 🞎 | 🞎 | 🞎 | 🞎 |
| 9. I feel competent with laboratory diagnostics within the HLIU | 🞎 | 🞎 | 🞎 | 🞎 | 🞎 |  | 🞎 | 🞎 | 🞎 | 🞎 | 🞎 |
| 10. I feel competent with placing a central line within the HLIU | 🞎 | 🞎 | 🞎 | 🞎 | 🞎 |  | 🞎 | 🞎 | 🞎 | 🞎 | 🞎 |
| 11. I feel competent with airway management within the HLIU | 🞎 | 🞎 | 🞎 | 🞎 | 🞎 |  | 🞎 | 🞎 | 🞎 | 🞎 | 🞎 |
| 12. I feel confident with the patient data management system | 🞎 | 🞎 | 🞎 | 🞎 | 🞎 |  | 🞎 | 🞎 | 🞎 | 🞎 | 🞎 |
| 13. Communication via the communication system within the HLIU works well | 🞎 | 🞎 | 🞎 | 🞎 | 🞎 |  | 🞎 | 🞎 | 🞎 | 🞎 | 🞎 |

Thank you
